# Factors influencing awareness of healthcare providers on maternal sepsis: a mixed-methods approach

**DOI:** 10.1186/s12889-019-6920-0

**Published:** 2019-06-03

**Authors:** Vanessa Brizuela, Mercedes Bonet, João Paulo Souza, Özge Tunçalp, Kasisomayajula Viswanath, Ana Langer

**Affiliations:** 1 UNDP/UNFPA/UNICEF/WHO/World Bank Special Programme of Research, Development and Research Training in Human Reproduction (HRP), Department of Reproductive Health and Research, World Health Organization, Avenue Appia 20, 1211 Geneva, Switzerland; 2 Department of Social Medicine, Ribeirao Preto Medical School, University of São Paulo, Ribeirão Preto, Brazil; 3 Department of Social and Behavioral Sciences, Harvard T.H. Chan School of Public Health, Boston, MA USA; 4 Department of Global Health and Population, Harvard T. H. Chan School of Public Health, Boston, MA USA; 5 Center for Community-Based Research, Dana-Farber Cancer Institute, Boston, MA USA

**Keywords:** Maternal sepsis, Awareness campaign, Knowledge, Enabling environment, Perception of disease, Multi-country study

## Abstract

**Background:**

An awareness campaign set to accompany the Global Maternal Sepsis Study (GLOSS) was launched in 2017. In order to better develop and evaluate the campaign, we sought to understand the factors that influence awareness of maternal sepsis by exploring healthcare providers’ knowledge, perception of enabling environments, and perception of severity of maternal sepsis.

**Methods:**

We used a mixed-methods approach that included 13 semi-structured interviews to GLOSS regional and country coordinators and 1555 surveys of providers working in GLOSS participating facilities. Directed content analysis and grounded theory were used for qualitative analysis, based on a framework including four overarching themes around maternal health conditions, determinants of maternal health, barriers and facilitators to sepsis identification and management, plus 24 additional sub-topics that emerged during the interviews. Descriptive statistics for frequencies and percentages were used for the quantitative analysis; significance was tested using Pearson χ^2^. Logistic regressions were performed to adjust for selected variables.

**Results:**

Analysis of interviews described limited availability of resources, poor quality of care, insufficient training and lack of protocols as some of the barriers to maternal sepsis identification and management. Analysis from the quantitative survey showed that while 92% of respondents had heard of maternal sepsis only 15% were able to correctly define it and 43% to correctly identify initial management. Provider confidence, perceived availability of resources and of a supportive environment were low (33%, 38%, and 48% respectively). Overall, the predictor that most explained awareness was training. Respondents from the survey and interviewees identified sepsis among the main conditions affecting women at their facilities.

**Conclusions:**

Awareness on maternal sepsis, while acknowledged as important, remains low. Healthcare providers need resources and support to feel confident about the correct identification and management of sepsis, as a prerequisite for the improvement of awareness of maternal sepsis. Similarly, providers need to know about maternal sepsis and its severity to understand the importance of reducing sepsis-related mortality and morbidity. Awareness raising campaigns can help bring neglected maternal health conditions, such as sepsis, to the forefront of global and local agendas.

**Electronic supplementary material:**

The online version of this article (10.1186/s12889-019-6920-0) contains supplementary material, which is available to authorized users.

## Background

In 2016 the Global Maternal and Neonatal Sepsis Initiative was launched with the goal to reduce deaths among women and newborns due to sepsis [1]. One of the first actions taken by this initiative was to obtain consensus on a new definition for maternal sepsis as “a life-threatening condition defined as organ dysfunction resulting from infection during pregnancy, childbirth, post-abortion, or postpartum period” [2]. Within this context, the Global Maternal Sepsis Study (GLOSS) and the accompanying maternal sepsis awareness campaign implemented in 2017 in 53 low-, middle-, and high-income countries across the world, were devised by the World Health Organization (WHO) to both assess the burden and management of maternal sepsis at a global level and to raise awareness on maternal sepsis among healthcare providers [3]. (See Table 1 for names of all GLOSS participating countries.)

**Table 1:** List of all countries that participated in GLOSS

Africa (13 countries)	Asia (9 countries)	Eastern Mediterranean (6 countries)	Europe (13 countries)	Latin America (11 countries)
Benin	Cambodia	Afghanistan	Belgium	Argentina
Burkina Faso	India	Egypt	Denmark	Bolivia
Cameroon	Mongolia	Lebanon	Italy	Brazil
Ethiopia	Myanmar	Morocco	Kazakhstan	Colombia
Ghana	Nepal	Pakistan	Kyrgyzstan	Ecuador
Kenya	Philippines	Sudan	Lithuania	Guatemala
Malawi	Sri Lanka		Republic of	Honduras
Mali	Thailand		Moldova	Mexico
Mozambique	Viet Nam		Netherlands	Nicaragua
Nigeria			Romania	Peru
Senegal			Slovakia	Uruguay
South Africa			Spain	
Zimbabwe			Tajikistan	
			United Kingdom	

One way in which awareness can be raised is through health communication campaigns. These have been used in multiple occasions to increase awareness among healthcare providers of a specific health topic, to improve knowledge or attitudes, or to prompt behaviour change toward health-related actions, such as promoting hand-washing or encouraging vaccination [4–8]. Despite the abundance of awareness campaigns, their impact on behaviour change has been somewhat limited [4, 9, 10]. Furthermore, while awareness raising efforts are commonplace, what is meant by awareness is oftentimes not consistently or well defined [11]. Awareness has been described at times as a precursor to action, at others as having heard of a specific issue, and most often measured through discrete assessments of increased knowledge alone [10, 12–14].

The goal of including an awareness campaign to accompany GLOSS was to sensitize the healthcare providers to the importance of maternal sepsis and improve their ability to identify warning signals, signs, and markers for infection and sepsis among pregnant or recently pregnant women.

This study sought to understand what factors influence healthcare provider awareness regarding the identification and management of maternal sepsis, including challenges and opportunities to increase awareness. Based on constructs from the behavioural sciences and health communication theories, for this study we defined awareness as a combination of concepts associated with knowledge, perception of enabling environments, and perception of severity of a condition that interrelate to sensitize people towards this problem [12, 13, 15–17].

## Methods

Study protocol for GLOSS has been published elsewhere. In short, GLOSS was a facility-based, prospective, one-week inception cohort study which was accompanied by an awareness campaign for healthcare providers in participating facilities. During 1 week between 28 November 2017 and 04 December 2017, all admitted or hospitalised women in a participating healthcare facility with suspected or confirmed infection during pregnancy through the 42nd day after end of pregnancy were eligible for inclusion in the study [3]. Women enrolled in the study were followed-up for up to 6 weeks until discharge, transfer outside the study area, or death. The campaign that accompanied the study was launched 3 weeks prior to data collection for a diverse audience of healthcare providers working in all participating facilities; the campaign continued throughout the entire study period and beyond. This study was led by WHO and coordinated through seven regional coordinators (one for each of the study regions) and a country coordinator in each participating country (53 total).

In order to obtain information on the factors that influenced provider awareness we used qualitative and quantitative methods through the use of semi-structured interviews and a survey, distributed primarily online, administered before campaign implementation [18–21]. The semi-structured interviews aimed to respond to the question “*what are the opportunities and challenges healthcare providers face with regards to maternal sepsis identification and management?*” while the surveys looked to answer “*what is the level of awareness on maternal sepsis identification and management, as expressed through knowledge of maternal sepsis and perception of work environments as enabling to correct identification and management?*” Semi-structured interviewing is one of the key techniques used in qualitative research, used to obtain personal opinions and experiences, allowing for elucidation of nuances, contradictions, and interpretations on a certain topic [18]. On the other hand, surveys are used frequently in quantitative research to collect information from a large population [21, 22]. Qualitative and quantitative data were collected between July and November 2017. Ethical approval for the entire study, including the awareness campaign was obtained from WHO’s Ethics Review Committee (protocol ID A65787).

We first describe the methods used to obtain the qualitative data through semi-structured interviews to key informants. We then describe the methods for collecting quantitative data through a survey of healthcare providers in the field.

### Qualitative – semi-structured interviews

We developed a guide for the interviews based on existing literature on maternal infections and sepsis, qualitative methodology, and our framework for conceptualising awareness to obtain information on barriers and facilitators, indicators and determinants, and perceptions of severity of maternal health conditions that might influence the identification and management of maternal sepsis [18, 20, 23, 24]. (See Additional file 1 for a copy of the interview guide.) We hypothesized that if interviewees did not see sepsis as a problem, their engagement with the study, and especially with the campaign, would be hindered. We also hypothesized that their perceptions of the causes of infections and sepsis might impact provider awareness on maternal sepsis as well as their ability to act. By asking about barriers and facilitators we expected to learn about existing challenges impacting provider awareness of maternal sepsis and potential opportunities that would help in developing the campaign.

We took a purposive sample of GLOSS regional and country coordinators for the interviews because they were respectively in charge of coordinating broad aspects of the study at the regional level and implementing the study on the ground. The sample was deemed appropriate given geographical and professional representation of all GLOSS regions and healthcare providers [25]. There were no refusals to being interviewed. VB conducted all the interviews in-person or over the phone, in English or Spanish, and knew one of the interviewees from prior collaborative work, while meeting all other interviewees for the first time during the interview. Interviews were conducted between July and August 2017. All the recorded interviews were transcribed verbatim and corroborated with notes taken during the interviewing. Translation of quotations used in this analysis was done by VB. Trustworthiness of data was ensured by interviewing persons fulfilling different roles in different countries/regions for GLOSS. Triangulation, through cross-checking data analysis between different researchers on the team (VB and MB) and the use of a mixed-method approach, was used to ensure robustness of the data [26].

### Quantitative – baseline surveys

We developed a 32-question survey to gather information on healthcare provider awareness on maternal sepsis, based on the literature and findings from the qualitative analysis [2, 23, 27–29]. The surveys were distributed primarily through an online platform, SurveyMonkey or Qualtrics, allowing us to reach a large audience. Online surveys were administered in eight languages: English, Spanish, Portuguese, French, Italian, Russian, Vietnamese, and Arabic. Paper-based copies were distributed at request in select countries in English, French, and Russian. (See Additional file 2 for a copy of the baseline survey.)

To ensure validity and reliability of the tool, i.e., the accuracy of the findings and the consistency of results, the survey was first piloted in English during 1 week in August 2017 [26]. For this, we asked regional coordinators for GLOSS to forward the survey to colleagues working in geographical areas that were excluded from the study.

The surveys gathered data on respondents’ knowledge, their perception of enabling environments, and their perception of severity of disease. The survey also asked respondents to identify main barriers to making correct and timely decisions with regards to recognising and managing maternal sepsis. Measures included Likert-scale, yes/no, and multiple-choice questions with discrete or unlimited answer options, and some fill-in response options. All questions were optional with some of them being conditional on responses to prior questions (e.g., question 10 asked “have you ever heard of the term 'maternal sepsis'”? and if the response was “no” then the following question would be automatically skipped). Allowing participants to recuse themselves from answering certain questions was essential to ethical considerations. Table 2 shows what variables from the survey were used to assess each of these three components, including the answers deemed correct for knowledge.

**Table 2:** Variables used in the analysis and answers deemed correct under knowledge

Knowledge	Perception of enabling environment	Perception of severity of disease
*Q10: Have you ever heard of the term “maternal sepsis”?* Answer: Yes	*Q4: How confident do you feel that you are capable of making the right decision in a case like the one above?*	*Q1: What are the main conditions causing death and disability among women during pregnancy and/or childbirth in your hospital? Check all that apply.*
*Q11: What two criteria best describe maternal sepsis?* Answer: infection & organ dysfunction	*Q5: How would you qualify the availability of resources in the facility where you work to help you make the right decisions?*	*Q14: How many women are affected by maternal sepsis in your facility every year? Give your best estimate (a whole number), given your experience in the facility.*
*Q2b/Q2a: What would be the first two things (this) woman should receive?* Answer: antibiotics & fluids^a^	*Q6: How supported do you feel by the facility in which you work to make the right decision in a case like the one above?*	*Q16: How many deliveries occur every year, on average, in your facility? Give your best estimate.*
	*Q17: Have you ever received specific training in how to manage women who present with signs of infection while pregnant, during childbirth, postpartum or post-abortion?*	

^a^Responses were deemed correct if respondents had accurately identified sepsis as the condition. Respondents were not penalized for not identifying the correct management if sepsis had not been selected as the condition

### Sampling strategy for the survey

We used a snowballing technique for survey dissemination. This was done primarily through regional and country coordinators who were asked to distribute and share the link to the online survey to the study focal persons at the participating facilities, who in turn were asked to disseminate among healthcare providers working in their facilities. A sample message was included in the emails that were sent out to facilitate the snowballing effect. Weekly reminders were sent through the online tool and via email. Targeted outreach was done with specific coordinators of countries with fewer responses. Surveys were collected between 29 September and 05 November 2017.

We included in the analysis responses from healthcare providers (i) that worked in a GLOSS participating facility and/or (ii) from a GLOSS participating country.^1^ We excluded all surveys completed by respondents that explicitly stated belonging from a geographical area not participating in GLOSS, and those by respondents stating their qualification as “other” without further clarification.

### Data analysis

For the qualitative analysis, we used concepts from directed content analysis and grounded theory [30]. We had a framework with four overarching themes and grounded theory allowed us to explore and allow the emergence of new topics that were grouped into the themes [31]. Atlas.ti (version 1.6.0 for Mac computers) was used for the interview analysis.

For the quantitative analysis, we used descriptive statistics to provide frequencies and percentages, and we tested for significance using Pearson χ^2^ test. We used logistic regression to adjust for selected variables referring to respondent and facilities’ characteristics which could explain differences in awareness, resulting from the prior test for significance. Potential confounders were: qualifications, age, years of experience, region, having received training in maternal sepsis, whether the facility was a public facility, or whether it was in an urban setting. We selected these confounders based on existing evidence that sociodemographic characteristics of respondents can influence knowledge and awareness on any given topic while making assumptions on facility characteristics which may explain differences in awareness among healthcare providers [5, 32–34]. We dichotomized the Likert-scale answers to allow for this analysis by assigning a 1 to the most favourable response (i.e., responding they felt *very confident* about being capable of making the right decision) and 0 to the combination of all the others (i.e., *somewhat confident, neutral, not very confident,* and *not confident at all*). Stata (version 14.2, College Station, TX) was used for quantitative data analysis.

## Results

We present the major findings from the qualitative and quantitative analysis separately.

### Qualitative – semi-structured interviews

A total of 13 country and regional coordinators for GLOSS were interviewed. Nine interviews were conducted in-person and four were conducted over the phone. Ten interviews were recorded; the other three were not because of technical difficulties (two cases) and participant refusal (one case). Three of the interviews were conducted in Spanish, the remaining ten were in English. The interviews lasted on average 40 min.

Six of the respondents were female and seven were male. There was at least one representative from every region participating in the study with up to three representatives from any single region. Six interviewees worked in research institutions in their home countries and five worked in hospitals. The remaining two interviewees worked either in WHO country offices or ministries of health.

During data analysis 24 subtopics emerged and were grouped into one of the four major themes included in our framework. Each of the four themes are described below including quotes from the interviews to support this analysis.

#### Theme I: severity of maternal health conditions

Conditions identified by interviewees from different regions largely responded to the main causes for maternal mortality and morbidity globally: post-partum haemorrhage, infections and sepsis, and pre-eclampsia/eclampsia, as well as abortion-related complications. Infections and sepsis were mentioned by 11 interviewees, representing all the study regions, as the conditions most frequently affecting women during pregnancy, childbirth, and postpartum or post-abortion. Equally, 11 interviewees mentioned post-partum haemorrhage and pre-eclampsia/eclampsia and other hypertensive disorders. Embolism, abortion-related complications, and indirect causes were mentioned by two, three, and seven interviewees respectively.

#### Theme II: determinants of maternal health

When asked about the perceived determinants of the maternal health conditions described before, with a specific emphasis on infections and sepsis, all of the interviewees identified the importance of social and cultural factors that impacted the health of the population and particularly women. Poverty, literacy, and nutrition were signalled as factors influencing maternal infections and this was regardless of the individual country’s level of development and wealth, as shown in the following quotes.

*To me, I think one of the important causes is about health literacy. I think people still don't have good knowledge about how to prevent themselves from infection, when it's the appropriate time to see a doctor. (Asia)*

*There are some regions in the country that are very poor. There is a very diverse population and areas with illiteracy where they speak a different language within the same country. (Latin America)*

*For sepsis during pregnancy, for me it has a lot to do with the nutrition conditions, eating, housing, overcrowding ( … ). (Latin America)*

#### Theme III: barriers to identifying and managing maternal sepsis

We also inquired about the challenges that healthcare providers faced in their facilities in correctly identifying and managing maternal sepsis. Among the emerging subtopics were: limited availability of resources and poor quality of services, difficulties with health system management, insufficient training, and lack of protocols. The following quotes support these findings.

*The level of care (provided) is not equal; the background of physicians and nurses is not the same and even inside the country the training levels are not the same. (Eastern Mediterranean)*

*The main barriers we see are related to training of healthcare providers and the high turnover of trained personnel. About 49% of the people we train [in emergency obstetric care] are no longer in the hospitals in which we trained them. (Latin America)*

*You need a higher level of antibiotic ( … ), there are very few, but they are with the manager, the clinical director of the hospital, so that means you have to write a letter to the clinical director, I have this patient, her name is this and this diagnosis, we did this test and now we need this. This has to go to the clinical director and then pharmacy, so it can take 2, 3, 4 days until we get the antibiotics, and this can be a real problem. Sometimes in the meantime the woman can die. (Africa)*

*Clearly for infections: the condition, the actual infrastructure, and the actual equipment. Sometimes, when you even look at the equipment in the hospital, you can easily see how and why people are getting infections, even in the hospitals. (Africa)*

#### Theme IV: facilitators to identifying and managing maternal sepsis

Nine interviewees focused on the motivation of some healthcare providers working in the facilities. Six interviewees were able to identify other systems that had been put in place in their facilities to record and provide guidance on what needed to be done to address infections and sepsis, such as the provision of team training. The next quotes support these claims.

*I think that the facilitators could be the motivation to save a woman’s life. (Africa)*

*Everyone wants to help the patients, wants to do the right thing, want to say that I am giving the same care as the best hospital [in a high-income country], for example. (Eastern Mediterranean)*

*Team training and simulation training, which has been very effective, certainly when dealing with obstetric emergencies. (Europe)*

*A very important thing that we have ( … ) are the committees for the prevention, surveillance and response of maternal and perinatal mortality and morbidity. (Latin America)*

Lastly, there was a large subtopic that emerged from many of the interviews relating to the need for more concerted efforts around infection and sepsis reduction, while there was also a suggestion of this being a good time for implementing new actions given existing initiatives to improve maternal health outcomes (such as the Ending Preventable Maternal Mortality Strategy, the WHO Resolution on Sepsis, and the Global Maternal and Neonatal Sepsis Initiative) [1, 35, 36].

Most interviewees mentioned the limited attention that infections and sepsis had up to date in the global and national agendas. This idea of sepsis not being visible, as exemplified by the following quotes, was integral to the development of the awareness campaign, as we were looking to bringing infections and sepsis to the forefront.

*To me sepsis is still not, it’s not on the agenda. (Latin America)*

*We know that yes, sepsis contributes to maternal morbidity, mortality, but there’s been no dedicated effort, no program to address the challenges posed by sepsis. None. (Africa)*

*Although we have free maternity care services in my country, there is no priority program for maternal sepsis, no government sector dealing with maternal sepsis ( … ) there is no awareness of sepsis. (Asia)*

### Quantitative – baseline survey

A total of 1555 surveys were completed: 1017 online and 538 paper-based from 48 out of the 53 eligible countries. Because of the methodology used for outreach (snowballing) and because we did not know what the total population of healthcare providers across all the participating facilities was, we could not estimate a response rate. We received an average of 30 responses per country (range 1–201). Thirty-three percent of survey responses were in Spanish, 32% were in English, and 19% in French; the remaining 16% were in the other languages. See Table 3 for a description of survey respondents and the facilities in which they worked.

**Table 3:** Characteristics of respondents and the facilities in which they worked as self-reported by respondents (*N* = 1555)^a^

Characteristic	N	%
Respondent characteristics		
Age (*N*=1398)		
<31	401	28.7
31–40	460	32.9
>40	537	38.4
Gender (*N*=1404)		
Male	345	24.6
Female	1059	75.4
Qualification (*N*=1402)		
Nurse/auxiliary nurse	245	17.5
Midwife	264	18.8
Physician	724	51.6
Resident	169	12.1
Years of experience (*N*=1327)		
<10	613	46.2
10–20	425	32.0
>20	289	21.8
Region^b^ (*N*= 1425)		
Africa (14 countries)	259	18.2
Asia (8 countries)	191	13.4
Eastern Mediterranean (5 countries)	246	17.3
Europe (11 countries)	238	16.7
Latin America (11 countries)	491	34.5
Facility characteristics^c^		
Location (*N*=1384)		
Urban	1288	93.1
Rural	96	6.9
Management (*N*=1391)		
Public	1150	82.7
Private/NGO/other	241	17.3

^a^Because respondents were allowed to leave questions unanswered, N for each question varied

^b^For each of the regions, the following countries are included: AFRICA: Benin, Burkina Faso, Cameroon, Chad, Ethiopia, Ghana, Kenya, Malawi, Mali, Mozambique, Nigeria, Senegal, South Africa, and Zimbabwe

ASIA: India, Mongolia, Myanmar, Nepal, Philippines, Sri Lanka, Thailand, and Viet Nam

EASTERN MEDITERRANEAN: Egypt, Lebanon, Morocco, Pakistan, and Sudan

EUROPE: Denmark, Italy, Kazakhstan, Kyrgyzstan, Lithuania, Republic of Moldova, Romania, Slovakia, Spain, Tajikistan, and United Kingdom

LATIN AMERICA: Argentina, Bolivia, Brazil, Colombia, Ecuador, Guatemala, Honduras, Mexico, Nicaragua, Peru, and Uruguay

^c^Out of the 632 facilities participating in GLOSS, 80% were in urban settings and 20% in non-urban areas; and 69% were public and 31% private/NGO/other. Description of the selection of participating facilities can be found elsewhere [3]

Following our definition of awareness, we present the most salient findings according to the three constructs that we included: knowledge on maternal sepsis, perception of enabling environments, and perception of severity of disease.

### Knowledge on maternal sepsis

Overall, 92% (1409/1529) of respondents said they had heard about maternal sepsis. However, only 15% (143/931) of respondents were able to specifically respond to the options *infection* and *organ dysfunction* when asked to identify the two criteria that best define maternal sepsis. Lastly, 43% (151/352) of respondents were able to correctly identify *antibiotics* and *fluids* from the options available as the two main treatments a woman with suspected infection/sepsis should initially receive.

After controlling for qualifications, age, years of experience, region, having received training in maternal sepsis, whether the facility was a public facility, or whether it was located in an urban setting, training and being from Europe were associated with increased odds of having heard of and identifying the correct criteria for maternal sepsis (aOR 4.97, 95% CI 2.65-9.34; aOR 3.10, 95% CI 1.08–8.91). Being a nurse or a midwife was associated with decreased odds of overall knowledge about maternal sepsis. There were no significant differences between regions with regards to identification of correct management of maternal sepsis. See Additional file 3 for the full models used in this analysis including all the variables.

### Perception of enabling environments

Overall, 33% (507/1525) of respondents said they felt very confident of making the right decision, 38% (574/1530) that resources were always available, and 48% (654/1367) that they felt very supported by the facility in which they worked. Fifty-four percent said they had received specific training in maternal sepsis.

After controlling for the same factors included in the previous model training was associated with increased odds of higher levels of confidence (aOR 2.06, 95% CI 1.59–2.68), perceived availability of resources (aOR 1.71, 95% CI 1.31–2.22), and feeling supported to make the right decisions (aOR 1.88, 95% CI 1.46–2.42). The odds of reporting availability of resources and of feeling supported were increased among nurses and respondents from Asia, Europe, and Latin America; being a nurse also increased perception of confidence. With the exception of nurses, there were no differences across other qualifications with regards to perceived enabling environments. See Additional file 4 for the full models used in this analysis including all the variables.

**Fig. 1:**
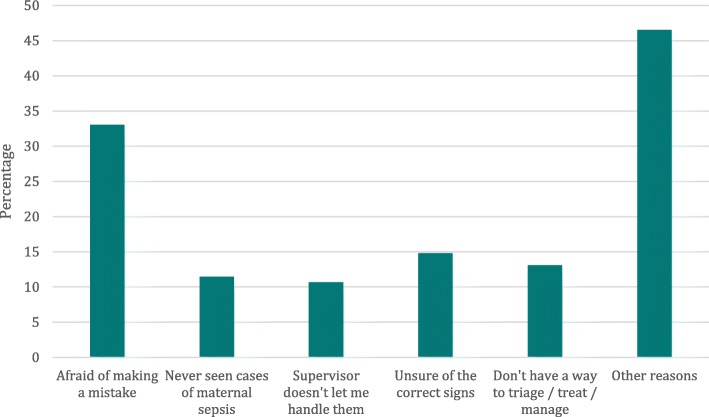
Barriers to making correct and timely decisions as reported by respondents (*N* = 1555). *Question allowed for selecting up to two response options*

Figure 1 shows the distribution of responses to existing barriers to making correct and timely decisions. Notably, 33% of respondents felt the greatest barrier was fear of making a mistake, followed by 15% indicating that they felt unsure they knew the correct signs to identify sepsis.

### Perceptions of the burden of disease

According to respondents, the main maternal health conditions affecting women were: haemorrhage (67%), pre-eclampsia/eclampsia (59%), and sepsis (51%). Respondents were asked to provide an estimated number of women affected by sepsis in their facilities every year. Since respondents were not asked to validate their responses against any reliable statistics, their responses do not represent accurate information, yet they provide insight on providers’ perceptions of the burden of sepsis and infections. On average, healthcare providers in Eastern Mediterranean were the ones most likely to estimate larger number of women affected by maternal sepsis compared to respondents in Asia.

## Discussion

We set out to explore the factors that influence healthcare providers’ awareness of maternal sepsis to better develop a campaign to accompany the Global Maternal Sepsis Study and later able to evaluate the impact and effectiveness of the campaign. We did this through semi-structured interviews with GLOSS regional and country coordinators and through a survey of healthcare providers working in participating facilities.

All respondents to the interviews and the survey considered infections as one of the main conditions affecting women during pregnancy, childbirth, and postpartum/post-abortion in their settings. In general, there was low knowledge of identification of maternal sepsis and low perception of confidence in making right decisions regarding maternal sepsis, with the exception of nurses who, while reporting low levels of overall knowledge, reported higher levels of confidence, perception of availability of resources, and feeling of support for making the right decisions, compared to physicians. Overall, the main factors influencing provider awareness were training and provider qualifications, and the region to which they belonged. Given the breadth of our survey sample which encompassed different cadres of providers at all levels of health care in 48 countries, we consider that our findings may apply to a larger population of healthcare providers.

Our study shows that training and certain professional qualifications are associated with higher levels of knowledge on maternal sepsis (knowledge was lower among nurses and midwives as compared to physicians and residents). Prior evidence supports the finding that training has the ability to influence provider awareness of sepsis identification and management, with some research also showing an effect on sepsis mortality [27, 28, 37]. Other studies have shown evidence that awareness campaigns can change provider knowledge [7, 8, 32, 38]. However, increased awareness was not always linked with increased or correct knowledge, which supports our broader definition of awareness that includes other factors (enabling environments and perception of severity of disease) [14].

In fact, we found that while nurses had overall low levels of knowledge, they reported positive perceptions of their environments. Respondents from Europe or Latin America reported positive perceptions of availability of resources and feeling of support yet low perceptions of confidence. The literature on health behaviour change supports the finding that environmental factors can influence provider awareness [16]. An implementation research study in Bangladesh that included training, an awareness campaign, and provision of resources found that each of the three components of the intervention impacted provider ability to respond to tuberculosis treatment in children [39]. An evaluation of a campaign aimed at changing tobacco-related attitudes and behaviour found that the context is critical in increasing awareness [40]. If healthcare providers are not offered the necessary tools or do not feel supported or confident about making the right decisions, they may not be able to improve their current clinical practices. Awareness and the consequent change in behaviour are made possible when, in addition to increased knowledge, healthcare providers work in an environment that facilitates their decision-making processes.

According to the data provided by our qualitative and quantitative assessment, we found that most interviewees and a majority of survey respondents reported infections as one of the main maternal health conditions, albeit a perception that sepsis was not yet on national or global agendas or as being somewhat “hidden.” Other studies have shown that people’s perception of disease severity impacts behaviour. A study looking at survey respondents’ ability to self-manage certain health conditions showed that the perception of severity of their condition impacted their ability to act on it [41]. Another study showed that perception of severity of disease greatly affected how respondents behaved regarding their risk or the importance they gave to said disease, while another meta-analysis of patient adherence to treatment showed that patient’s perception of the severity of disease affected their ability to follow a treatment regimen [42, 43].

Our study has some limitations. First, interviews were held only in English or Spanish, which restricted the sample to those proficient in those languages. However, given representatives from all GLOSS participating regions were interviewed, we believe that the finding from the qualitative data analysis is generalizable to the population of focus: healthcare providers working in facilities where women with suspected or confirmed infections or sepsis are hospitalized. Similarly, although the survey was professionally translated and double-checked by technical experts fluent in all languages, the fidelity of these translations and the nuances of meaning in different countries were not assessed. Second, pilot testing of the survey was done only in English and in its online version. Potential issues with validity of the tool, comprehension, and flow of the questions in other languages could not be identified, as well as opinions from people with limited internet connectivity. Third, while we were able to offer the survey in paper-based format as a means to overcome internet access, this meant that skip patterns and response options which were automatic on the online tool were not always followed according to instructions on paper. Fourth, the use of snowballing as a technique to disseminate the survey has inherent potential response biases and these might impact the results from the quantitative component.

## Conclusions

Awareness on maternal sepsis remains low. Correct identification and management of maternal sepsis is deficient and the environments in which healthcare providers work are not optimal for them to accurately understand and respond to maternal sepsis. The factors that mostly influence awareness of healthcare providers on maternal sepsis are training and qualifications. Specific training on sepsis has the potential to significantly influence healthcare providers’ knowledge of maternal sepsis while qualifications impact the perception of how enabling the environment is. Because enabling work environments can affect behaviour, healthcare facilities should encourage confidence in making the right decisions as well as foster supportive work environments, and ensure resources are available for maternal sepsis correct identification and management.

Awareness raising campaigns can help bring specific maternal health conditions, such as infections and sepsis, to the forefront. A campaign aimed at increasing awareness among providers in multiple countries has the potential to improve awareness for sepsis identification and management. This study provided a more in-depth understanding of the factors affecting provider awareness of maternal sepsis allowing for the development of a campaign, as well as a sound basis upon which to evaluate campaign impact and effectiveness. The GLOSS campaign, which has now been evaluated (soon to be published) sought to raise awareness by increasing knowledge, improving provider perceptions of their environments, and adjust their perception of the severity of maternal sepsis to the reality in their contexts.

## Additional files

Additional file 1: Interview guide. (DOCX 21 kb)

Additional file 2: Baseline survey. (DOCX 23 kb)

Additional file 3: Logistic regression models used for the knowledge component (DOCX 19 kb)

Additional file 4: Logistic regression models used for the enabling environment component. (DOCX 21 kb)

## Abbreviations

ERC: Ethics Review Committee
GLOSS: Global Maternal Sepsis Study
NGO: Non-Governmental Organization
RHR: Reproductive Health and Research
UNDP: United Nations Development Programme
UNFPA: United Nations Population Fund
UNICEF: United Nations Children’s Fund
USAID: United States Agency for International Development
WHO: World Health Organization

## Footnotes

1 At time of baseline survey collection some GLOSS participating countries were still finalizing the selection of facilities.
