# Supplementary material for: Factors influencing awareness of healthcare providers on maternal sepsis: a mixed-methods approach
Source: BMC Public Health. 2019 Jun 3;19:683. doi: 10.1186/s12889-019-6920-0 (PMC6547516; doi:10.1186/s12889-019-6920-0)
Supplement: Supplementary file 1 — Interview guide. (DOCX 21 kb) [file 12889_2019_6920_MOESM1_ESM.docx]

**Global Maternal Sepsis Awareness Campaign**

This interview is part of the activities set forth for the Global Maternal Sepsis Study (GLOSS) that is being conducted in approximately 50 countries across the globe.

This study is being coordinated by the World Health Organization and (INSERT COORDINATING INSTITUTION IN COUNTRY) in your country. The goal of this global study is to assess the burden and management of complicated infections during pregnancy, childbirth, postpartum, or the post-abortion period. An important component of this study is a campaign to be deployed in participating healthcare facilities to increase provider awareness around this issue.

**This interview is confidential and its purpose is to gather your thoughts and opinions about maternal and neonatal sepsis identification and management.**  This interview should not take longer than 45 minutes. There are no right or wrong answers, but rather this is an opportunity to hear your thoughts about the topic as a (REGIONAL COORDINATOR/COUNTRY COORDINATOR) participating in this study. If you agree to this, I will record the conversation so that I can later remember what we talked about. I will likely be taking notes as well while we speak. I will also ask you to complete and sign an informed consent form. You will keep a signed copy with you.

Thanks in advance for agreeing to participate in this activity.

**To start off, a few questions about yourself …**

1. Sex: (OBSERVE)

|  | Female |  |
| --- | --- | --- |
|  | Male |  |

1. What country are you from? (KNOWN AHEAD OF TIME)
2. What is your professional background? How long have you been working in this field?
3. What is your job title?
4. What is your experience with multi-country studies?
5. What is your experience participating in awareness campaigns?

**I WILL NOW ASK YOU ABOUT SOME MATERNAL AND NEONATAL HEALTH CONDITIONS IN THE GEOGRAPHICAL AREA WHERE YOU WORK…**

1. What are the main health conditions affecting women during pregnancy and childbirth in your workplace? (PROBE: in your REGION/COUNTRY/HOSPITAL)
2. What about neonates?
3. What do you think are the main factors that influence these conditions?
4. What, if anything, is being done to address these issues?
5. (IF THEY HAVE MENTIONED INFECTIONS): Let’s focus on infections/sepsis.
6. What are the main infections affecting women in your area? In your hospital?
7. How does your area (PROBE GEOGRAPHICAL AREA) deal with women presenting with this condition? And your hospital?

(IF THEY HAVE NOT MENTIONED INFECTIONS):

1. Could you tell me about infections/sepsis affecting women in your area (PROBE: GEOGRAPHICAL)? And in your hospital?
2. What are the main infections affecting women in your area? In your hospital?
3. How does hospital deal with women presenting with this condition?
4. What are the main barriers, if any, that providers face when encountered with cases of maternal sepsis?
5. What about any facilitators? (PROBE: WHAT TYPE OF SUPPORT, IF ANY, DO THEY RECEIVE FROM THEIR HOSPITALS/MANAGEMENT?)
6. What about barriers and/or facilitators faced by healthcare facilities?
7. How does your (REGION/COUNTRY/HOSPITAL) respond to cases of maternal and neonatal sepsis?
8. What, if any, are the guidelines or procedures to record cases of sepsis?
9. Could you tell me about any initiatives that you might know of for dealing with maternal sepsis in your (GEOGRAPHICAL AREA/HOSPITAL)?
10. Lastly, as part of this study, we are planning on sending out a survey to providers working in participating hospitals. The plan is to make this an online survey. If you agree, I would be sending you a copy of this survey for your feedback and input. (WAIT FOR ANSWER ON WHETHER THEY AGREE TO THIS OR NOT).
11. What is the feasibility of asking providers in your area or hospital to complete a survey that requires access to the internet on a computer or smartphone?
12. What are your thoughts on developing a campaign for increasing awareness on maternal sepsis (PROBE: WILL THIS BE EFFECTIVE IN PROVIDING INFORMATION? WHERE DO PROVIDERS GET MOST OF THEIR MEDICAL INFORMATION/TRAINING?)
13. Is there anything else you would like to add?

**Thanks for participating!**
