# Supplementary material for: Factors influencing awareness of healthcare providers on maternal sepsis: a mixed-methods approach
Source: BMC Public Health. 2019 Jun 3;19:683. doi: 10.1186/s12889-019-6920-0 (PMC6547516; doi:10.1186/s12889-019-6920-0)
Supplement: Supplementary file 2 — Baseline survey. (DOCX 23 kb) [file 12889_2019_6920_MOESM2_ESM.docx]

**Global Maternal Sepsis Study – GLOSS Campaign Survey**

This online survey is part of the activities set forth for a global study on maternal morbidity and mortality. This study is being conducted in approximately 50 countries across the globe, including your own, and it is coordinated by the World Health Organization and the healthcare facility where you work.

As part of this study, we want to learn more from healthcare providers about how you identify and manage women with complications during pregnancy, childbirth, postpartum, or post- abortion. The survey includes a number of questions on your knowledge, attitudes, and practices around maternal and neonatal health. This is not a test; this is an opportunity to let us know your thoughts and experience on the topic as a healthcare provider in one of the hospitals participating in the study.

This survey is voluntary and your answers will be kept confidential, and you can choose whether to leave some questions unanswered. General information about you, your position, and geographical location will be collected to help us categorize respondents only, but will not be used to identify you in particular. You are free to provide this information at the end of the survey.

After the study, and only if you agree, a second online survey will be sent to you via email. For this reason, we will ask you to provide an email address so that we can ensure delivery of the second survey. You will be free to decide to participate in this second survey too. Results of these surveys will be published in a peer-reviewed journal without attributing responses to any specific person or institution.

**The completion of this survey implies your consent to participate.**

If you have any question about the survey please contact Ms Vanessa Brizuela.

***I. Knowledge and attitudes***

The following questions will ask that you respond according to your current role, competences, and skills depending on your training and background. That is, according to these, you may be the person triaging, prescribing, diagnosing, treating. Bear this in mind when responding.

1. What are the **main** conditions causing death and disability among women during pregnancy and/or childbirth in your hospital? Check all that apply [abortion-related complications, chronic/pre-existing disease, embolism, haemorrhage, infection/sepsis, pre-eclampsia/eclampsia, other: please specify]
2. Case vignettes:

Case A: A 25-year-old 32- week pregnant woman comes to your facility brought by a family member saying she is feeling unwell. Her companion reports that she seems a bit disoriented and feverish. Without any further diagnostic testing or triaging:

Case B: A recently pregnant woman comes to your facility complaining that she has abdominal pain and shortness of breath. Without any further diagnostic testing or triaging:

- 1. What would you **first** think could be causing her to feel this way? Choose from the following list [abortion-related complications, embolism, haemorrhage, infection/sepsis, pre-eclampsia/eclampsia, other]
  2. What would be **the first two things** this woman should receive? [antibiotics, blood transfusion, body fluid culture, fetal monitoring, fluids, haematology/biochemistry laboratory, other antimicrobials (i.e. antimalarials, ART), other laboratory test, other medication, oxygen, physical exam, urine output measurement, other]

1. How confident do you feel that you are capable of making the right decision in a case like the one above? [very confident, somewhat confident, neutral, not too confident, not confident at all]
2. How would you qualify the availability of resources in the facility where you work to help you make the right decisions? [always available, somewhat available, neutral, not always available, not available at all].
3. How supported do you feel by the facility in which you work to make the right decision in a case like the one above? [very supported, somewhat supported, neutral, not very supported, unsupported].
4. How well does this statement describe your facility: “The facility where I work doesn’t let me handle cases like the one described above.” [very well, somewhat well, indifferent, somewhat incorrectly, completely incorrectly]
5. Of the following, which do you think are the greatest barriers in making a right, and timely decision in your facility? Check **up to two options**. [I’m afraid of making a mistake, I’ve never seen cases like these, my supervisor doesn’t let me make them, not sure I know the correct signs, we don’t have a way to triage/treat/manage cases like these in my hospital, other]
6. Does the hospital you work in have protocols in place for dealing with cases like the one described above? [yes/no/don’t know]

DO NOT ALLOW GOING BACK AFTER THIS QUESTION

1. Have you ever heard of the term maternal sepsis? [yes/no]
2. If yes, how did you hear about this? Check all that apply [pre-service training, in-service training, public health campaign, colleagues, media (TV/radio/newspaper), other: please specify].
3. What two criteria best describe *maternal sepsis*? Check **two options** [abnormal white cell count, altered mental status, elevated heart rate, excessively rapid respiration, fever, infection, low blood pressure, organ dysfunction, other]
4. What supplies/commodities are essential to effectively **identify** sepsis among women during pregnancy, childbirth, postpartum or post-abortion? Check all that apply [blood culture, blood pressure apparatus, diagnostic imaging, laboratory (haematology/biochemistry), rapid test for infectious disease, serum lactate measurement, thermometer, urine output measurement, other]
5. What supplies/commodities are essential to effectively **manage** sepsis in women during pregnancy, childbirth, postpartum or post-abortion? Check all that apply [antibiotics, blood transfusions, fluids, intensive care/high-dependency unit, other antimicrobials (e.g. antimalarials, ART), oxygen, urine output measurement, other]

***II. Context***

1. How many women are affected by maternal sepsis in your facility every year? Give your best estimate (a whole number), given your experience in the facility
2. How many neonates are affected by neonatal sepsis in the first week of life in your facility? Give your best estimate (a whole number), given your experience in the facility.
3. How many deliveries occur every year, on average, in your facility? Give your best estimate (a whole number).
4. Have you ever received specific training in how to manage women who present with signs of infection while pregnant, during childbirth, postpartum or post-abortion? [yes/no/can’t remember]

***III. Personal information***

Remember! These data are collected for categorization purposes only. Your information is confidential and you will not be identified in any future publications on this study.

1. Age range
2. Gender: [male, female, other]
3. Qualification: [nurse, midwife, physician/medical doctor, resident/physician in training, community health worker, social worker, other: please specify].
4. Years of work experience in current setting: [years|months]
5. Total years of work experience (since completing your training):
6. Place of work:
7. Location (of current or main place of work): [urban/rural]
8. Name of facility & address
9. Facility type (of current or main place of work): [Clinic, Health centre, Maternity hospital, Regional/Provincial hospital, District hospital, Other hospital , other]
10. Facility management (of current or main place of work): [private, public, social insurance, NGO, other]
11. Did you participate in this year’s World Sepsis Congress Spotlight on Maternal and Neonatal Sepsis (held on 12 September 2017)? [yes/no]

***IV. Future contacts***

1. *The global maternal study and awareness campaign would like to contact you at a future date for a follow-up on this survey. If you agree to being contacted again, please provide us with your email address.*

*Your contact details will be securely stored by the WHO staff person working on the study for one year. You can contact us to modify or suppress your information at any time. To do so, please contact Ms Vanessa Brizuela.*

- *I agree.*
- *I do not agree*

Please provide us with your email address

**Thank you very much for participating in this survey! Your responses are extremely valuable to us in our efforts to improve the health of women and newborns.**
