# Supplementary material for: Factors influencing awareness of healthcare providers on maternal sepsis: a mixed-methods approach
Source: BMC Public Health. 2019 Jun 3;19:683. doi: 10.1186/s12889-019-6920-0 (PMC6547516; doi:10.1186/s12889-019-6920-0)
Supplement: Supplementary file 3 — Logistic regression models used for the knowledge component (DOCX 19 kb) [file 12889_2019_6920_MOESM3_ESM.docx]

**Association between respondent and facility characteristics and respondent knowledge of maternal sepsis**

| **Predictor** | ***Had heard of maternal sepsis*** *(N=1,528)* | | ***Correctly identified the two criteria to define maternal sepsis*** *(N=931)* | | ***Correctly identified management of sepsis when maternal sepsis was suspected*** *(N=352)* | |
| --- | --- | --- | --- | --- | --- | --- |
|  | % Yes | aOR [CI] | % Yes | aOR [CI] | % Yes | aOR [CI] |
| *Overall* | 92.2 |  | 15.4 |  | 42.9 |  |
| *Qualification* |  |  |  |  |  |  |
| Nurse | 91.4 | 0.45 [0.19-1.04] | 3.1 | 0.16 [ 0.06-0.43] | 24.6 | 0.17 [0.08-0.40] |
| Midwife | 73.8 | 0.21 [0.10-0.41] | 9.1 | 0.53 [0.24-1.17] | 33.3 | 0.52 [0.20-1.40] |
| Physicians | 97.4 | 1 | 22.1 | 1 | 48.0 | 1 |
| Resident | 97.0 | 1.87 [0.40-8.80] | 27.7 | 1.54 [0.85-2.81] | 65.5 | 1.78 [0.79-3.99] |
| *Age* |  |  |  |  |  |  |
| <31 | 91.5 | 0.74 [0.31-1.73] | 15.5 | 0.77 [0.43-1.38] | 43.3 | 0.98 [0.46-2.11] |
| 31-40 | 91.5 | 1 | 18.4 | 1 | 48.5 | 1 |
| >40 | 92.3 | 2.07 [1.00 -4.29] | 17.0 | 0.67 [0.34-1.31] | 42.9 | 1. 03 [0.44-2.41] |
| *Years of experience* |  |  |  |  |  |  |
| <10 | 95.9 | 2.50 [1.11-5.65] | 19.1 | 0.93 [0.49-1.73] | 48.1 | 0.75 [0.33-1.67] |
| 10-20 | 89.5 | 1 | 15.5 | 1 | 42.2 | 1 |
| >20 | 90.6 | 0.49 [0.22-1.06] | 16.8 | 1.16 [0.60-2.27] | 44.2 | 2.17 [0.87-5.38] |
| *Region* |  |  |  |  |  |  |
| Africa | 93.8 | 1 | 14.2 | 1 | 51.6 | 1 |
| Asia | 94.1 | 2.01 [0.74-5.52] | 6.3 | 0.66 [0.25-1.74] | 13.3 | 0.18 [0.04-0.78] |
| Eastern Mediterranean | 67.5 | 0.49 [0.24-1.00] | 3.7 | 0.24 [0.05-1.09] | 16.1 | 0.14 [0.03-0.59] |
| Europe^†^ | 97.1 | 3.10 [1.08-8.91] | 35.8 | 3.50 [1.79-6.86] | 33.3 | 0.43 [0.17-1.07] |
| Latin America | 98.6 | 3.96 [1.44-10.91] | 19.6 | 1.64 [0.89-3.02] | 59.7 | 1.81 [0.88-3.70] |
| *Training (yes)* | 97.5 | 4.97 [2.65-9.34] | 20.7 | 1.66 [1.08-2.57] | 55.4 | 2.11 [1.15-3.84] |
| *Public facility (yes)* | 90.8 | 0.41 [0.16-1.03] | 18.3 | 1.08 [0.57-2.03] | 44.8 | 0.58 [0.27-1.27] |
| *Urban facility (yes)* | 92.2 | 1.00 [0.39-2.56] | 17.5 | 1.83 [0.68-4.97] | 47.4 | 9.66 [1.78-52.33] |
| *aOR: adjusted odds ratio; CI: confidence interval* | | | | | | |
| *^†^Includes countries in Central Asia (Kazakhstan, Kyrgyzstan, and Tajikistan)* | | | | | | |
| *Adjusted for respondent qualifications, age, years of experience, region, whether they’d received specific training in maternal infections and sepsis, whether they worked in a public facility, and whether the facility in which they worked was located in an urban environment.* | | | | | | |
