# Supplementary material for: Factors influencing awareness of healthcare providers on maternal sepsis: a mixed-methods approach
Source: BMC Public Health. 2019 Jun 3;19:683. doi: 10.1186/s12889-019-6920-0 (PMC6547516; doi:10.1186/s12889-019-6920-0)
Supplement: Supplementary file 4 — Logistic regression models used for the enabling environment component. (DOCX 21 kb) [file 12889_2019_6920_MOESM4_ESM.docx]

**Association between respondent and facility characteristics and respondent perception of enabling environments in making right decisions for identifying and managing maternal sepsis**

| **Predictor** | ***Felt very confident of making the right decision*** *(N=1,524)* | | ***Reported resources were always available to make the right decision*** *(N=1,529)* | | ***Felt very supported by the facility in which they worked to make the right decision*** *(N=1,366)* | |
| --- | --- | --- | --- | --- | --- | --- |
|  | % Yes | aOR [CI] | % Yes | aOR [CI] | % Yes | aOR [CI] |
| *Overall* | 32.6 |  | 36.9 |  | 42.0 |  |
| *Qualification* |  |  |  |  |  |  |
| Nurse | 42.5 | 1.52 [1.07-2.16] | 59.9 | 2.76 [1.91-4.00] | 68.2 | 2.54 [1.76-3.66] |
| Midwife | 29.0 | 0.87 [0.59-1.27] | 30.4 | 1.27 [0.86-1.88] | 35.1 | 0.96 [0.66-1.40] |
| Physician | 33.9 | 1 | 34.3 | 1 | 46.4 | 1 |
| Resident | 27.1 | 0.99 [0.64-1.55] | 30.2 | 1.15 [0.74-1.78] | 43.8 | 1.19 [0.78-1.82] |
| *Age* |  |  |  |  |  |  |
| <31 | 24.1 | 0.62 [0.43-0.89] | 37.4 | 1.15 [0.79-1.67] | 46.5 | 0.80 [0.56-1.14] |
| 31-40 | 34.2 | 1 | 31.5 | 1 | 44.4 | 1 |
| >40 | 40.1 | 1.06 [0.74-1.51] | 42.6 | 1.11 [0.76-1.62] | 51.3 | 1.08 [0.75-1.55] |
| *Years of experience* |  |  |  |  |  |  |
| <10 | 28.2 | 0.77 [0.54-1.10] | 34.1 | 0.76 [0.52-1.11] | 45.1 | 0.81 [0.57-1.16] |
| 10-20 | 36.8 | 1 | 36.4 | 1 | 47.5 | 1 |
| >20 | 44.7 | 1.32 [0.91-1.90] | 52.5 | 1.59 [1.09-2.33] | 57.8 | 1.15 [0.80-1.67] |
| *Region* |  |  |  |  |  |  |
| Africa | 43.0 | 1 | 26.4 | 1 | 36.4 | 1 |
| Asia | 39.2 | 0.89 [0.56-1.42] | 41.5 | 2.78 [1.68-4.61] | 50.6 | 1.76 [1.09-2.84] |
| Eastern Mediterranean | 23.6 | 0.59 [0.37-0.93] | 21.0 | 1.16 [0.70-1.93] | 32.0 | 1.10 [0.69-1.77] |
| Europe^†^ | 25.2 | 0.45 [0.29-0.69] | 47.4 | 4.12 [2.64-6.43] | 57.4 | 3.39 [2.23-5.16] |
| Latin America | 35.5 | 0.80 [0.57-1.13] | 45.1 | 2.57 [1.74-3.80] | 54.8 | 2.12 [1.48-3.05] |
| *Training (yes)* | 41.1 | 2.06 [1.59-2.68] | 44.1 | 1.71 [1.31-2.22] | 54.5 | 1.88 [1.46-2.42] |
| *Public facility (yes)* | 33.1 | 1.11 [0.79-1.55] | 31.2 | 0.22 [0.16-0.32] | 42.3 | 0.26 [0.19-0.37] |
| *Urban facility (yes)* | 33.1 | 0.56 [0.35-0.90] | 38.5 | 1.37 [0.82-2.30] | 48.5 | 0.84 [0.52-1.37] |
| *aOR: adjusted odds ratio; CI: confidence interval* | | | | | | |
| *^†^Includes countries in Central Asia (Kazakhstan, Kyrgyzstan, and Tajikistan)* | | | | | | |
| *Adjusted for respondent qualifications, age, years of experience, region, whether they’d received specific training in maternal infections and sepsis, whether they worked in a public facility, and whether the facility in which they worked was located in an urban environment.* | | | | | | |
